# Supplementary figures and images for: Phe-Gly motifs drive fibrillization of TDP-43’s prion-like domain condensates
Source: PLoS Biol. 2021 Apr 28;19(4):e3001198. doi: 10.1371/journal.pbio.3001198 (PMC8109789; doi:10.1371/journal.pbio.3001198)

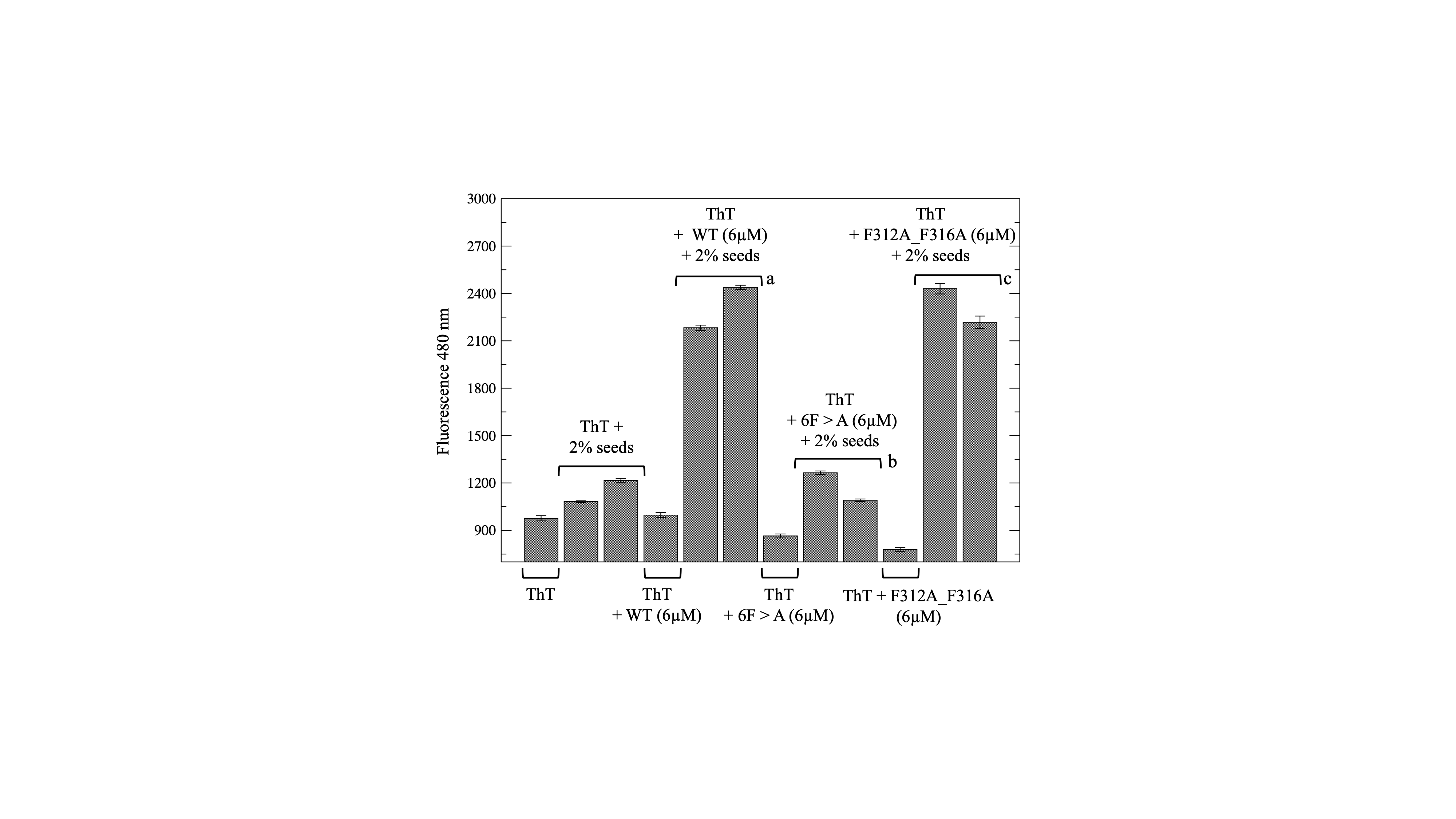

Supplement: S1 Fig — Cross-seeding in the polymerization into amyloid fibrils of the WT with respect to a double mutant, F313A+F316A, and a hexa-mutated construct, F276A+F283A+F289A+F367A+F397A+F401A, was studied by ThT binding assays. The distinct columns represent the following samples: column 1 is a blank, consisting of ThT (40 μM) in 1 mM CD3COOD (blank), and columns 2 and 3 are 2 independent samples consisting of 2% (0.12 μM) of seeds (amyloid fibrils packed in the SSNMR rotor). The next 3 columns (4, 5, and 6) correspond to 3 independent samples from the WT TDP-43 PrLD (1 without and 2 with 2% seeds). In columns 7, 8, and 9, the results for the hexa-mutant (F276A+F283A+F289A+F367A+F397A+F401A, denoted as 6F > A) are presented (1 without and 2 with 2% seeds). Finally, columns 10, 11, and 12 correspond to the double mutant F313A+F316A (1 without and 2 with 2% seeds). ThT is at 40 μM in all instances, and the final protein concentration is always 6 μM. Seeds from the WT cannot induce amyloid formation in the 6F > A mutant protein, as revealed by the negligible ThT enhancement (p-values between “a” and “b” or between “b” and “c” <0.0001). In contrast, F313A+F316A is efficiently cross-seeded in the presence of 2% of seeds (p-value between “a” and “c” of 0.8224). Each independent sample is measured 5 times, and the corresponding numerical data can be found in S1 Data. PrLD, prion-like domain; TDP-43, Transactive response DNA-binding Protein of 43 kDa; ThT, Thioflavin T; WT, wild-type. (TIFF) [file pbio.3001198.s001.tiff]
